# Modeling Stem Cell Induction Processes

**DOI:** 10.1371/journal.pone.0060240

**Published:** 2013-05-08

**Authors:** Filipe Grácio, Joaquim Cabral, Bruce Tidor

**Affiliations:** 1 Institute for Biotechnology and Bioengineering (IBB), Centre for Biological and Chemical Engineering, Instituto Superior Técnico, Lisboa, Portugal; 2 Computer Science and Artificial Intelligence Laboratory, Massachusetts Institute of Technology, Cambridge, Massachusetts, United States of America; 3 Department of Biological Engineering, Massachusetts Institute of Technology, Cambridge, Massachusetts, United States of America; 4 Department of Electrical Engineering and Computer Science, Massachusetts Institute of Technology, Cambridge, Massachusetts, United States of America; University of Kansas Medical Center, United States of America

## Abstract

Technology for converting human cells to pluripotent stem cell using induction processes has the potential to revolutionize regenerative medicine. However, the production of these so called iPS cells is still quite inefficient and may be dominated by stochastic effects. In this work we build mass-action models of the core regulatory elements controlling stem cell induction and maintenance. The models include not only the network of transcription factors *NANOG*, *OCT4*, *SOX2*, but also important epigenetic regulatory features of DNA methylation and histone modification. We show that the network topology reported in the literature is consistent with the observed experimental behavior of bistability and inducibility. Based on simulations of stem cell generation protocols, and in particular focusing on changes in epigenetic cellular states, we show that cooperative and independent reaction mechanisms have experimentally identifiable differences in the dynamics of reprogramming, and we analyze such differences and their biological basis. It had been argued that stochastic and elite models of stem cell generation represent distinct fundamental mechanisms. Work presented here suggests an alternative possibility that they represent differences in the amount of information we have about the distribution of cellular states before and during reprogramming protocols. We show further that unpredictability and variation in reprogramming decreases as the cell progresses along the induction process, and that identifiable groups of cells with elite-seeming behavior can come about by a stochastic process. Finally we show how different mechanisms and kinetic properties impact the prospects of improving the efficiency of iPS cell generation protocols.

## Introduction

Stem cells are in an undifferentiated state of development and have the potential to progressively differentiate to become adult somatic cells. Following the classification and nomenclature suggested by Jaenisch and Young [1] there are cells that can form all the lineages of the body – these are called pluripotent stem cells – and there are cells that form cell types of only a single lineage – multipotent stem cells. Because pluripotent stem cells can become any cell type in the body, they have strong potential to be transformative in a range of medical applications. In fact, the use and application of stem cells in the field of regenerative medicine are now well documented [2]–[5]. Possible advantages and flexibility of embryonic stem cells are countered by a variety of concerns resulting in different degrees of regulation around the world that can restrict possible applications as well as available funding [6]. Thus, it was a significant breakthrough when, in 2006, it was reported that partially differentiated mouse cells had been reprogrammed to become induced pluripotent stem cells (iPSCs) [7] and, more significantly, in 2007 adult human fibroblasts were also reprogrammed to become human iPSCs [8]. The experimental protocol made use of viral vectors transfected into fibroblasts driving the exogenous expression of 4 transcription factors (*OCT4*, *SOX2*, *c-MYC*, and *KLF4*). These experimental protocols, given sufficient time of exogenous gene expression and posterior selection, produced a subpopulation of iPSC. It has been recognized that human iPSCs have chromatin and gene expression patterns that are very similar to embryonic stem cells [9], and in functional essays show pluripotency potential [10]–[12]. There have since been several other reports of related methods with variations to obtain iPSC (see two excellent recent reviews by Patel and Young [13] and by Amabile and Meissner [14]). It has also been recognized that induced pluripotent stem cells, like embryonic stem cells, are of great potential for regenerative medicine [5], in particular due to the possibility of matching the cell donor to the recipient (who can be the same individual). Some of this excitement has been tempered by some difficulties with iPSC including potentially deleterious mutations that may need to be overcome [15].

Despite the relative success of the technology to induce pluripotency, a persistent issue is the low level of efficiency of the protocols. In fact, the average efficiency has been reported to be close to 0.5% with some protocols having efficiencies in the order of 1 iPSC cell transformed for each 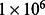 adult cells that were subjected to the experimental conditions [14]. Other reports put the average efficiency of the process somewhere between 0.02% and 0.002% [16]. Low reported efficiencies indicate that there is room for improvement in stem cell generation protocols [3].

Mechanistic understanding of cellular reprogramming is still at an early stage of understanding. Nevertheless effort has been put into trying to find ways of improving the efficiency of reprogramming; there has been work studying the effect of varying transcription factor concentrations and their relative quantities in the efficiency of the process [16], as well as work investigating the effect of the addition of small molecules to the protocols [17], [18]. Despite the progress enabled, the underlying processes that lead to the observed effects are only beginning to be explained. In fact, arguably one barrier to progress in improving the efficiency of these protocols is that, although the end point is relatively well characterized, little is known about the detailed mechanisms that underlie the necessary changes for reprogramming to be completed. Among some of the outstanding problems, is the intriguing observation that subjecting a large populations of cells to the same experimental conditions results in different outcomes for different cells with only a subset being reprogrammed. This outcome variability has created a debate about the nature of the reprogramming process. Yamanaka proposed two distinct hypothesis for stem cell generation that he called "stochastic" and "elite" [19]. The elite hypothesis posits that the low efficiency in reprogramming comes from only some cells having the potential to reprogram and upon induction doing so; whereas the stochastic hypothesis posits that all cells have potential to reprogram, and low efficiency is explained by random events that cause some to reprogram and others not to, under the particular conditions of the experiment. The distinction seems important and bears consequences if one wants to increase the efficiency of reprogramming protocols. In one case attention should focus on identifying, amplifying, or producing more elite cells capable of being induced, whereas in the other case attention should be on improving protocols to shepherd more cells along the induction pathway.

There has recently been valuable work in building dynamic models of the core circuitry of cellular pluripotency. Despite the important progress such work represents, the models are still in an early stage of development – as indeed would be expected from the early stage of kinetic and mechanistic understanding of the biological foundations of cellular reprogramming. For example Hanna and co-workers, alongside their experimental work, modeled the reprogramming process as a two-state, single-step stochastic transition from adult to reprogrammed cell [20]. However, such a model cannot account for the influence of biological features like regulatory network interactions. A step in that direction is a two-species model for the genes OCT4 and NANOG that uses ordinary differential equations to simulate their interaction as mutual regulators [21]. The model is used to show consistency with the experimental observations of bimodality of the distribution of gene expression. However, the reported model does not have two steady states that one might expect to correspond to an adult state, and a reprogrammed state. Other work focuses on lineage specification, and includes the gene SOX2 and its interactions with OCT4 and NANOG as well as other genes. It gives an account of how the system of regulatory interactions can form the foundation of cellular differentiation [22].

Despite representing important progress, these works do not not capture several important physical properties of the system under study. Namely, they do not allow for explicit modeling of the reprogramming protocols (inducing adult cells with transcription factors), they do not include epigenetic events that are known to be important to stem cell reprogramming, and they cannot be used to study the low copy number of biological molecules (most notably DNA strands) that strongly influence the stochastic nature of the system. Furthermore, the previously published kinetic models do not allow for computational interrogation of the influence of the rates of particular steps, or different biological mechanisms, which are the focus of this work. We contribute to the field by building a mathematical model that includes some of the known details of the mechanism of regulation and addresses some of the main questions important for understanding and improving induced reprogramming processes. Such a model is not to be viewed as a finished product but rather as a step in the development of ever more accurate models of the process of induction. We build a model of stem cell reprogramming and the first question we address with the model is simple but essential: if we model the interactions and relations between species for which there is documented experimental evidence, does the outcome for the model match that observed experimentally? A positive result indicates that known events, species and interactions are sufficient to account for the observed biology. There are three particular properties that we wish to reproduce: (1) Bistability, *i.e.* the existence of two states of gene expression, one corresponding to an adult cell with low expression level of the pluripotency associated genes, and another state corresponding to the stem cell, of high levels of expression of these same genes; (2) Inducibility, *i.e.* the capability of transitioning from one state to the other under conditions that correspond to the experimental protocols of stem cell induction; and (3) Variability, *i.e.*, the feature that under the same conditions different cells in a population have different outcomes. Here we focus on the distribution of times at which individual cells reprogram, if they reprogram at all. The results of simulations demonstrate that current biological knowledge is sufficient to account for observed behavior.

Next, we investigate how the pattern of kinetic steps affects the dynamics and distribution of reprogramming outcomes. Preferred pathways and slow steps lead to bottlenecks that can produce signatures in the distribution of reprogramming times. Moreover, cellular treatments or perturbations that accelerate rate limiting steps can be effective at improving reprogramming efficiency and need not be applied continuously. Because populations tend to build up just prior to slow reaction steps, a brief pulse applied at an appropriate time can lead to dramatic increases in reprogramming yields. Furthermore, patterns of bottlenecks could exist such that different sub-populations of cells reprogram at vastly different rates. Finally, whether one views the behavior as elite or stochastic is partially semantic, as discussed in more detail herein.

## Methods

### The Model Core

To study and understand the processes involved in stem cell reprogramming, we built several simplified models of the process. We first describe the core shared by all the models and how that leads to our base model, and then proceed to introduce subsequent models and the differences among them.

**Figure 1:**
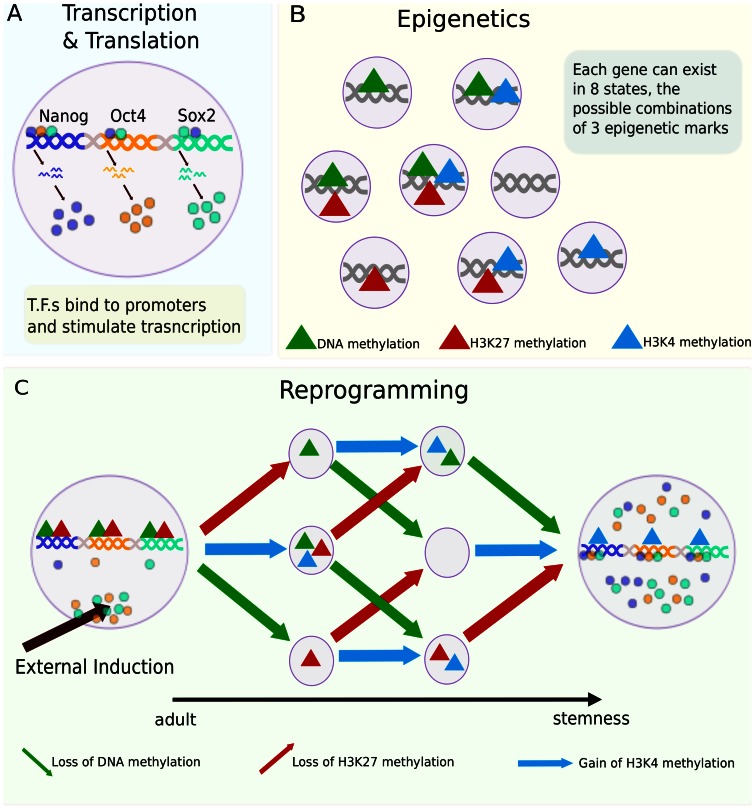
Cellular states and reprogramming. (A) Our model includes promoters capable of binding the three transcription factors *NANOG*, *OCT4,* and *SOX2*. Transcription from the corresponding genes produces mRNA, and translation of mRNA leads to protein. (B) Each promoter also has 3 epigenetic variables, creating 8 possible states for each of the genes. (C) Reprogramming protocols consist of starting with low transcription rates of these transcription factor genes and a particular combination of epigenetic marks, and through external induction (addition of transcription factors) progressing through modifications in the cell that lead to the actively transcribing state with the alternate combination of epigenetic marks. We simulate this reprogramming process with different models that explore the consequences of different mechanisms and kinetics.

The models include the promoter regions of the core genes in the circuitry responsible for pluripotency: *OCT4*, *SOX2*, and *NANOG* [21], [23], [24]. These genes encode transcription factors and experimental evidence shows each of the promoters can have each of the factors (OCT4, SOX2 and NANOG) bound to it or not [1], [23], [25], [26]. Based on promoter state the genes produce mRNA, and protein gene product is produced by translation at a rate proportional to the quantity of mRNA (Figure 1A). We also consider each gene to be characterized by the presence or absence of three epigenetic features known to be important for pluripotency [27], namely, DNA methylation, histone-3 K27 (H3K27) methylation, and histone-3 K4 (H3K4) methylation (Figure 1B). Actively transcribing pluripotency genes are known to be characterized by low DNA methylation, low H3K27 methylation, and high H3K4 methylation [14], [24], [28], [29]. These features are incorporated in our model by assigning that epigenetic state the highest transcription rate for pluripotency genes; all other promoter states have a low basal transcription rate.

The modeled effect of transcription factor proteins is two fold. First, when bound to promoters they facilitate transcription (the transcription rate is higher when all three factors are bound). Second, consistent with current literature (although the mechanism is not known) they mediate epigenetic changes [30], [31] involved in gaining pluripotency: *i.e.*, they facilitate the loss of DNA methylation, loss of H3K27 methylation, and gain of H3K4 methylation by making these changes more likely when the factors are bound. Figure 1C represents the two end states and six intermediate states in which each promoter in the system can exist, and the transformations required to move from one state to the other. On the left side the promoters have only DNA methylation and H3K27 methylation, with no transcription factors bound to them and low transcription rate; upon external induction, the epigenetic state of the genes changes (represented in the figure in unknown order and multiple pathways) until only the H3K4 methylation is present and the pluripotency genes have the transcription factors bound to them and high endogenous transcription rates (far right of figure). Each promoter changes states, independently of the others, such that each promoter in the system (two for each gene, six in total) can be in any of the states represented in the figure regardless of the state of the others.

The model also includes the dimerization of the protein NANOG to create the active form of the transcription factor [32]. We further incorporate the reported cooperativity in binding of the OCT4 and SOX2 transcription factors [33], [34] by making the factors bind more easily and dissociate less easily when the other factor is already bound. The model includes two copies of each of the genes and degradation of all the mRNA and protein species.

### Model Variants

In order to study the effects of mechanistic differences in iPSC generation protocols, we built several model variants that reflect different biological possibilities and system behavior with respect to the epigenetic marks. We focused on epigenetic changes because they are known to be important limiting factors in reprogramming [35].

We start with a base model, which we call Independent Equiprobable, and then create variants by introducing different reaction rates that reflect different assumptions about the mechanistic and kinetic aspects of reprogramming. We alter the reactions rates only for the *NANOG* gene, which is activated for the cell to be considered a stem cell – both experimentally [20] and in this model – and therefore the one that we track in this work.

#### Independent equiprobable

**Figure 2:**
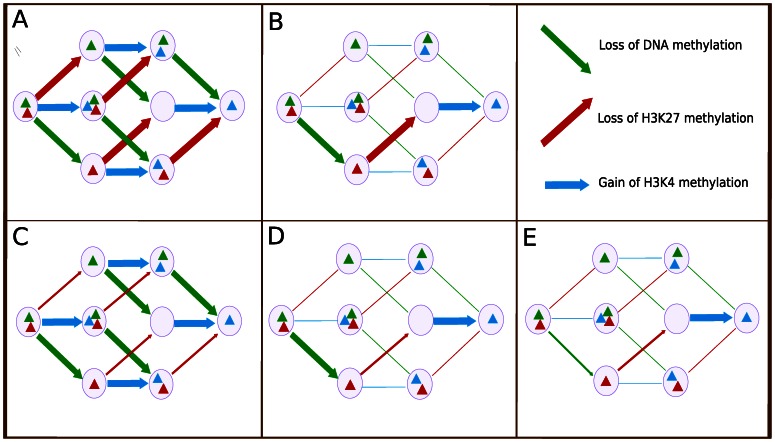
Graphic depiction of different models. (A) Independent Equiprobable model. (B) Cooperative Equiprobable model. (C) Independent 1 Slow Step model. (D) Cooperative 1 Slow Step model. (E) Cooperative 2 Slow Steps model. In each of the panels the thickness of the lines represents the rate of the corresponding reaction.

The Independent Equiprobable model reflects the hypothesis that the epigenetic reactions happen independently and at similar rates (hence equiprobable). This is achieved by making all the rate parameters of all the epigenetic changes equal. The consequence is that at any point, any of the epigenetic changes is equally likely to occur independently of the others (Figure 2A). This model serves as our base model; the other models will be constructed starting from this through changing rate constants.

#### Cooperative equiprobable

An alternative biological mechanism is that there is a specific order in which the reactions must occur. This corresponds to a cooperative mechanism (for example, a situation in which the enzyme responsible for K4 methylation can only bind the histone after it has lost the K27 methylation). To create this model we started from the Independent Equiprobable model and reduced the rate constants to create the necessary cooperativity. In particular, for H3K4 methylation is favored once H3K27 demethylation occurs, and H3K27 demethylation is favored once DNA demethylation has occurred. Thus, there is a single preferred path involving DNA demethylation then H3K27 demethylation and finally H3K4 methylation (Figure 2B). The goal here is to study the effect of having a preferred path, not to claim the in stem cell reprogramming this is the preferred one.

#### Independent 1 slow step

To model the case in which particular reactions are less likely (slower) than others without a preferred path, we changed the Independent Equiprobable model to create one with a reaction step – loss of H3K27 methylation – that is independent of the others and ten times slower. This is the Independent 1 slow step model. Because loss of H3K27 methylation can occur from any of the states with any combination of the other two epigenetic marks, four overall arrows in the reaction network are actually slowed (Figure 2C).

#### Cooperative 1 slow step

In a similar manner we changed the Cooperative Equiprobable model by decreasing by twenty-fold the loss of H3K27 methylation compared to related reactions (loss of DNA methylation and gain of H3K4 methylation). This produces a preferred path with a bottleneck (Figure 2D).

#### Cooperative 2 slow steps

Finally, to reflect the case of multiple bottlenecks along the way to reprogramming, we changed the Cooperative Equiprobable model to introduce two slow steps – loss of DNA methylation and loss of H3K27 methylation – when compared to the remaining step of gain of H3K4 methylation. This produces a preferred pathway with consecutive bottlenecks (Figure 2E).

It should be noted that the set of variants is useful to explore the behavior of this general reaction scheme. The variations are not exclusive and can be generalized and adapted to other epigenetic changes of gene activation circuits that might be considered important.

### Implementation

All of the models were built in MATLAB 2008b (Mathworks Inc; Natick, MA) with the SimBiology toolbox. To simulate the models we used the MATLAB-compatible KronckerBio toolbox previously developed in this lab. In the absence of experimentally measured parameters for our system, parametrization of the model was initially taken from previous theoretical and experimental work. Model parameters were then adjusted so that our system produced the experimental observation of reasonable amounts of mRNA and protein, as well as two steady states. Table 1 provides a comparison between the range of parameters found in references [36]–[46], and the ones used here for transcription, translation, mRNA degradation, protein degradation, and transcription factor binding and dissociation. The reactions that can happen in the model (binding and dissociation of transcription factors, gain and loss of epigenetic traits, transcription, translation, dimerization and dissociation of NANOG dimer, and degradation of species) follow mass-action kinetics. This means that the rate 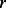 of the reaction 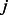 is defined by the equation,
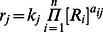
(1)where 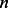 is the number of species in the model, 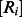 are the concentrations of species, 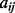 is an exponent (0, 1 or 2) representing the order of involvement of the species 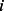 in reaction 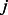, and 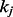 is the rate constant of reaction 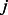. Models were thus constructed so that they could be represented as a series of ordinary differential equations (ODEs) for each species with terms corresponding to the reactions that either consume or create it. To exemplify how we build the equations to simulate the model, we here give an example for a particular state of the promoter of the Nanog gene. The state does not have DNA methylation, does have H3K27 methylation, does not have H3K4 methylation, and has only the transcription factor SOX2 bound to it. The concentration of that species is represented by the symbol 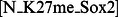. The letter 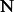 representing the fact that it is the NANOG promoter, the 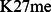 representing the H3K27 methylation, and 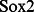 representing the transcription factor SOX2 being bound to the promoter. The reactions in which species 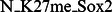 can be formed, and those that can consume it, are given in Table 2.

**Table 1:** Table of parameter values.

Parameter	Values found in literature	Values used here
Transcription	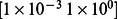	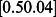
Translation	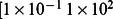	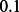
mRNA degradation	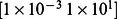	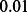
Protein degradation	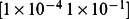	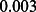
TF dissociation	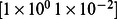	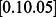
TF binding	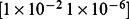	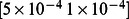

One column shows a range of representative values present in the literature, and the other column shows the value used in this work. All parameters, except last row, are of first order reactions and therefore have units of 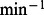. The TF binding constant has units of 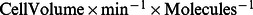, with cell volume assumed to be in the range measured for mammalian cells [58].

**Table 2:** Table illustration the reactions that a promoter state can be formed or consumed from.

Reactant1	Reactant2	Product1	Product2	Param.	Value
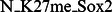	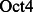	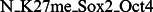		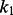	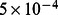
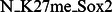	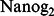	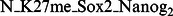		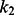	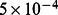
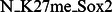		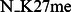	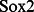	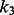	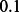
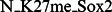		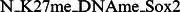		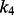	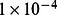
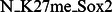		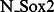		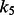	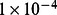
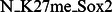		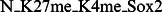		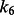	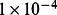
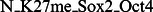		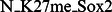	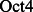	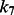	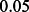
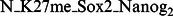		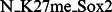	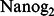	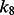	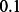
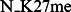	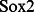	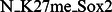		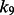	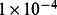
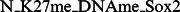		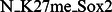		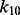	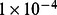
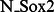		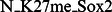		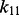	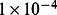
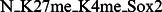		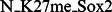		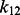	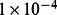

The example is for the 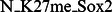 species. Parameters of first order reactions have units of 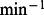 and parameters for second order reactions have units of 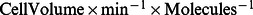.

The species is consumed by 6 reactions and is formed by 6 other reactions. It is consumed by (1) OCT4 binding to it, (2) NANOG dimer binding to it, (3) SOX2 dissociating from it, (4) gaining DNA methylation, (5) losing H3K27 methylation, and (6) gaining H3K4 methylation. All these reactions create different species and consume the original species. This promoter state is also generated from other species by specific reactions and modifications. Namely it is formed by, (7) OCT4 dissociating from 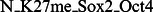, (8) the NANOG dimer dissociating from 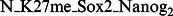, (9) 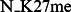 and SOX2 binding together, (10) 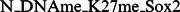 losing its DNA methylation, (11) 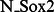 the acquiring H3K27 methylation, (12) 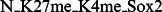 losing H3K4 methylation. From these reactions that produce or consume 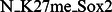, we write the appropriate ordinary differential equation for the time dependent variation in the concentration of the species.
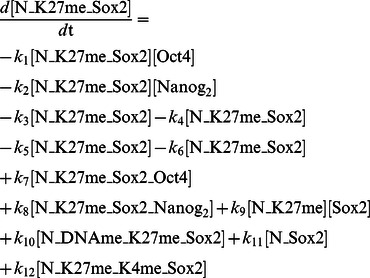



The equation contains each of the depletion or production terms corresponding to the reactions in Table 2. This equation, and all others like it for the other species of the model, is then simulated using deterministic mass-action kinetics by means of the ode15s routine in MATLAB.

The model was also implemented in a stochastic form and simulated using Gillespie's stochastic simulation algorithm (SSA), which we implemented and used except where otherwise noted. The Gillespie SSA is a methodology to simulate the behavior due to the stochasticity of random times of biochemical reactions. The algorithm works by considering molecule counts (as opposed to concentrations) and assuming that each biochemical reaction can be described as a Poisson process with exponentially distributed waiting time to occur. Once the algorithm is initialized, a random number generation is used to draw from the appropriate distributions to obtain a time for the next reaction and the identity of that reaction (relative probabilities are calculated based on their rates); once the reaction is selected, the time step and the number of molecules of the reagents and products are updated appropriately. Details for the implementation of the algorithm can be found in the original work [47].

The first simulation method described (using ODEs) is deterministic and only provides results that are the average for identical experiments that are in reality non-deterministic. The second simulation method (the Gillespie SSA) is non-deterministic and, by using several simulations (that differ by the sequence of random numbers generated), is used to investigate statistical properties of the simulated model system. In cases where the differences between single simulations is important, such stochastic transitions between states, or when the counts of molecules are low (for example, two copies of DNA strands per cell and their respective promoter states), then this methodology is appropriate.

All models are provided as MATLAB files as Supplementary Material.

### Stochastic Simulations of Standard Induction

Simulations of induction processes were carried out, for each of the models, using 500 independent simulations (corresponding to 500 independent cells in culture). Each cell, unless otherwise noted, started the simulation with no internal transcription factors and with all promoters in the epigenetic state with only DNA methylation and H3K27 methylation (the left-hand side of Figure 1C). At time 0 an exogenous source of both OCT4 and SOX2 mRNA was added, and the simulation proceeded until a time limit specific for each model. We then analyzed the data and measured the time at which changes of epigenetic state occur and the time at which the cells changed to the reprogrammed state (defined by the concentration of NANOG dimer going above a threshold value of 1200 molecules per cell).

### Stochastic Simulations of Accelerating Induction

To study the effect of accelerating reactions in the reprogramming protocols, we followed a similar procedure to the one described above. Each cell started the simulation with zero concentration of transcription factors, and with all promoters in the epigenetic state with only DNA methylation and H3K27 methylation (the left-hand side of Figure 1C). We wanted to study the effect of two variables: which reaction to accelerate and the time to do so. We ran ten different experiments, each experiment consisted of selecting a reaction to accelerate and a time for intervention. We chose two possible reactions to accelerate: loss of DNA methylation or loss of H3K27 methylation. The times of intervention corresponded to intervals with a duration of 10% of the total protocol time. The acceleration was applied between 0 and 10%, 10–20%, 20–30%, 30–40%, 40–50% (note that 2 reactions at 5 possible times, result in the 10 different experiments for each model). The acceleration consisted of making the selected reaction 10 times more likely (faster) than in the original setting. We ran 100 simulation per trial and we used three models: Independent 1 Slow Step, Cooperative 1 Slow Step, and Cooperative 2 Slow Steps.

### Population Equilibration and Simulation

To test the effect of cell state on induction outcome, we ran induction simulations with a prior stochastic equilibration step. The equilibration of the Independent Equiprobable model consisted of simulating a group of 1500 cells without induction factors, for enough time that the distribution of states reached an equilibrium distribution (that is, the percentage of cells in each state remained constant over time).

To study the effect of the initial state of the cell prior to reprogramming on the reprogramming dynamics of all mechanisms, for each model we populated each of the *NANOG* promoter states individually with at least 100 cells, then ran the induction simulation with exogenous source of OCT4 and SOX2 (identically to what is described previously), and captured the reprogramming time and other events for each cell.

## Results and Discussion

We built a series of models to describe the reprogramming of adult cells to iPS cells and used these models to gain insight into stem cell induction processes. We focus on the effects of three genes (*NANOG*, *SOX2*, and *OCT4*) and three epigenetic features (DNA methylation, H3K27 methylation, and H3K4 methylation). We will first demonstrate that the base model we built has features that are observed experimentally. We then use the set of model variants to perform stochastic simulations to study features of induction.

### Bistability, Inducibility, and Variability

The first question we addressed is whether the simple topology implemented from experimental evidence can reproduce the three features of bistability, inducibility, and variability. This question is important to establish whether known biology is itself sufficient to account for observed features, or whether additional mechanisms are required.

**Figure 3:**
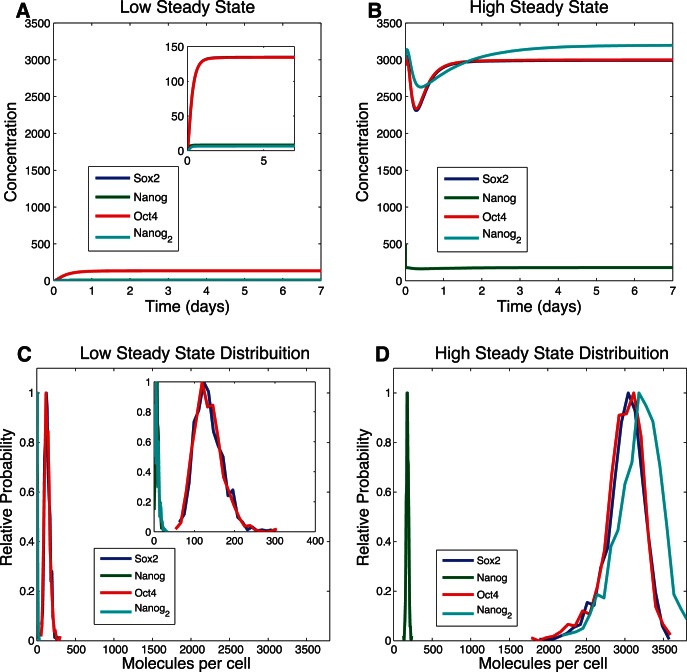
Observation of bistability. (A) Result of simulating the ODE model with initial conditions corresponding to low concentrations of transcription factor protein and mRNA (close to uninduced state). (B) Result of simulating the ODE model with initial conditions corresponding to high concentrations of transcription factor protein and mRNA (close to induced state). (C) Distribution of final values for the proteins of the model with initial conditions in low state (D) Distribution of final values for the proteins of the model with initial conditions in are state. Results for panels C and D are for 500 stochastic simulations for the Independent Equiprobable model.

To probe for bistability, we performed two different integrations of the model's ODEs with two different sets of initial conditions. One set of initial conditions had low levels of mRNA and protein for all three transcription factors, and had the promoters for all genes set with the epigenetic marks characteristic of the uninduced state (DNA methylated, H3K27 methylated, and H3K4 demethylated); the other set of initial conditions had high levels of mRNA and protein for all three transcription factors, and had the promoters for all three genes set with the epigenetic marks characteristic of the induced state (DNA demethylated, H3K27 demethylated, and H3K4 methylated). Bistability was demonstrated by the model converging to two different steady states that depended on the initial condition (Figure 3A and B). That bistability is a property of this complex network of interactions shows not only that the model captures some of the essential qualitative features of the real system, but also that the current level of biological knowledge is consistent with the experimental observation.

To further explore the model properties contributing to bistability in this model, we studied the effects of different biological mechanisms. Specifically we investigated (1) epigenetic cooperativity that couples epigenetic states to changes in transcription factor binding [30], [31], (2) dimerization of the transcription factor NANOG [32], and (3) cooperative binding of OCT4 and SOX2 [33], [34]. The model has all three properties, and it is bistable. We explored models having combinations of subsets these of features and tested for bistability. When bistability was lost, a quick manual search over parameter space was performed to investigate whether bistability could easily be recovered with different parameters but without changing cooperativity.

The results, in Table 3, indicate that bistability in our models relies principally on the cooperativity between epigenetics and transcription factor binding. All models with this property can achieve bistability, although minor re-parametrization may be required (rows 2 and 6). The data also show that NANOG dimerization plays a role: when this property is removed from the full model (moving from row 1 to row 6 in Table 3) the immediate effect is a loss of bistability. Finally, cooperative binding of OCT4 and SOX2 in the models does not play a significant role: its loss from row 1 to row 5 caused no loss of bistability. It is also interesting to note that epigenetic coperativity alone can be a mechanism for bistability, without requiring dimerization cooperativity or cooperative binding of transcription factors. The data indicate that mechanisms can act together to bring about bistability and multiple contributing mechanisms may lead to greater robustness of bistability across varying parameters.

**Table 3:** Bistability studies.

Epig. Coop. (a)	Nanog Dim. (b)	OCT4/SOX2 Coop. (c)	Bistability (d)
1	1	1	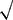
1	0	0	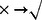
0	1	0	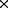
0	0	1	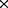
1	1	0	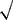
1	0	1	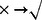
0	1	1	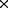

Each column represents a different feature of the model: (a) Epigenetic cooperativity with the Transcription factors; (b) the dimerization of the transcription factor NANOG; (c) the cooperative binding between OCT4 and SOX2; and (d) the observation of bistability. Each row represents a different model variation. The presence or absence of a feature in a model is marked respectively by a 1 or a 0. In the bistability column an
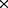
represents no observed bistability, and a 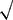 represents observed bistability; an arrow from one to the other represents a successful change in parameters resulting in bistability.

The above results were all obtained with the deterministic ODE version of each model. An important goal of the current study is to use stochastic simulation methodology to explore cell-to-cell variability in reprogramming dynamics, as such variability is a feature of experimentally observed reprogramming protocols. Deterministic simulations produce the same behavior when started with the same initial conditions and once they reach a steady state they remain there if unperturbed. Stochastic simulations use random event generation to simulate the non-deterministic characteristics of the timing of chemical reaction events. Thus, in a stochastic framework, which can be a more realistic treatment of biochemical systems, bistability can lie along a continuum from transient to persistent. For the purpose of iPSC generation protocols, persistent bistability that switches state only when stimulated by induction protocols would be preferable. Here we have made stochastic simulations and compared bistability properties to those in the ODE framework. We simulated stochastic dynamics for the Independent Equiprobable model with the Gillespie Stochastic Simulation algorithm [47] for initial conditions corresponding to the uninduced state (500 times), and for initial conditions corresponding to the induced state (also 500 times). The distribution of outcomes for each of the two initial conditions is shown in Figure 3C and D. As expected, the data show that the model has two different distributions that depend on the initial conditions, with simulations beginning near the induced or uninduced state generally remaining there.

**Figure 4:**
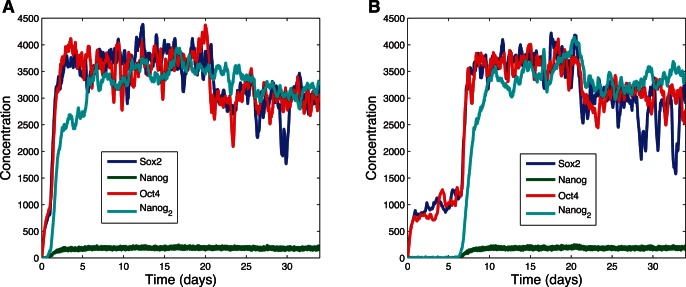
Demonstration of inducibility. Two examples of stochastic simulations of induction for the Independent Equiprobable model. At time 0 exogenous expression of SOX2 and OCT4 was begun. (A) In one simulation the internal circuit changed state at day 1. (B) For a second simulation the change occurred at day 6. In this demonstration, at day 20 for both cases, the external induction was removed.

Next, we investigated whether the bistable system could be induced from the low steady state to the high steady state by adding an exogenous source of SOX2 and OCT4. Simulations were carried out with mRNA for these genes produced at a constant rate to emulate viral- or plasmid-based induction, as is done experimentally. The results of hundreds of stochastic simulations demonstrate all simulations can eventually reach the induced state and remain there, although there was great variance in the time required for induction, ranging from about one day to several weeks of simulated time. The trajectories for two stochastic induction simulations are given in Figure 4. At 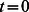 the induction protocol began. In one of the illustrated simulations, the system changes state at day one (Figure 4A), and in the other the change happens at day six (Figure 4B). It is important to note that at day 20, for both cases, the external induction is removed (hence the slight drop of transcription factor levels), but the system remains in the high state.

Thus, the simulation results display marked cell-to-cell variability. Beyond the small difference expected when stochastic dynamics are simulated, dramatic variation in induction times were observed, with some cells reprogramming in about a day and others taking several weeks, although each cell was modeled identically.

### Relationship between Pathway Kinetics and Reprogramming Dynamics

Mechanistic information describing the detailed progression of steps of cellular reprogramming is still emerging, and this has led to different abstractions regarding the nature of reprogramming, including the proposed stochastic versus elite framework [19]. It would be valuable to develop a better understanding of the connections between the mechanism and functional properties of the pluripotency circuitry and how they might be better probed experimentally. Next we report simulations exploring relationships between reprogramming pathway kinetic and mechanistic properties, and overall dynamics of reprogramming.

#### Event timing carries information about mechanism and progress of cellular reprogramming

**Figure 5:**
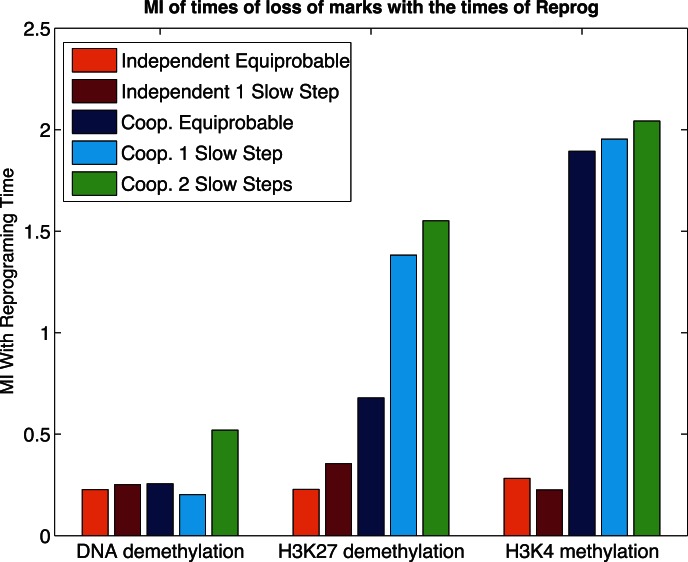
Mutual information. On the horizontal axis are represented the 3 measured variables (times of the different events). On the vertical axis is the mutual information each of the variables has with the reprogramming time.

What is the relationship between events along the reprogramming pathway and the overall reprogramming time? How does this relationship change in the context of different biological mechanisms? To study this we chose to focus on the times at which three types of events happen: the time at which the first of the two copies of the *NANOG* promoter loses DNA methylation, the time at which the first of the two copies of the *NANOG* promoter loses H3K27 methylation, and the time at which the first of the two copies of the *NANOG* promoter gains H3K4 methylation. For each model, we simulated 500 cells subject to external induction with OCT4 and SOX2, acquired the times at which these events occurred, and from the ensemble of simulations computed the mutual information [48], [49] between the times of each of these three events and the time of reprogramming. The results are given in Figure 5.

Starting with the Independent Equiprobable model (orange bars), the results show that each of the events has essentially the same amount of mutual information with the final reprogramming time and that amount is relatively low. This implies that none of the observations is more informative than the others about the final reprogramming time. For the independent model, knowing that a single event has happened implies nothing about the other events and therefore provides little information about when all the necessary events will finally occur. By contrast the Cooperative Equiprobable model (dark blue) shows a clear progression of increasing mutual information with the final reprogramming time that corresponds to the preferred path of epigenetic changes. Thus, in a cooperative mechanism, as the cell progresses to the final state, the time at which events happen increases the information and predictability about the time of the final event. This is important because it suggests that, in cooperative mechanisms, stochasticity (unpredictability) is reduced along the reprogramming process, and that the information of a single event is dependent on where that event stands in the chain of cooperative reactions – crucially that information is preserved if we measure only that event. We should note that this progressive increase of information in the cooperative mechanisms does not come from the step being closer to the final time. In fact, if the times at which the events of H3K27 methylation happened were just a constant waiting time after loss of DNA methylation, then the latter would not add any information at all. Rather, occurrence of the first event reduces variability in the time of reprogramming.

Comparing the Cooperative Equiprobable model (dark blue) with the Cooperative 1 Slow Step one (light blue), shows a big increase in the mutual information at the point of loss of H3K27 methylation – which is precisely the slow step. The results indicate that the slowest step in the process is also the one whose occurrence most reduces the uncertainty about the final event timing. Essentially, the slow step increases variability about when reprogramming will occur; later steps are faster and thus have a tighter relationship with the final reprogramming time.

The results from the Cooperative with 2 Slow Steps model (green) show that the occurrence of the first slow step (DNA demethylation) adds more information when compared with the same step in other models. As a slow step, there is greater variability in its timing, so its occurrence significantly reduces the variability in the time of reprogramming. Because there is a further slow step yet to be achieved, the mutual information with the final reprogramming time is of intermediate magnitude. Because this is the only model in which this step is slow, the magnitude is larger than in the other models.

Because reactions are reversible, an acquired epigenetic state can be lost. Therefore, the importance of – and information contained in – the "first time" the state changes is dependent on the stability of the change. If an acquired epigenetic mark is highly prone to loss, then having gained it for the first time is not very informative about the progress of the cell over the pathway of necessary transformations. In our models, under conditions of induction, the back reactions are less likely than moving forward (the exact reaction rate depends on the amounts of the reagents).

#### Probability curves uncover kinetic aspects of process

**Figure 6:**
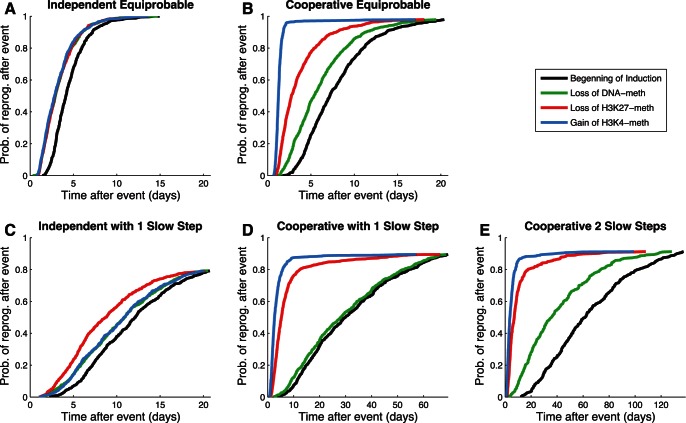
Probability curves for reprogramming. The horizontal axis represents time after an epigenetic event. The vertical axis is the cumulative probability of observing reprogramming given that an event has happened for the first time. Each of the lines represents the occurrence of events in the legend. Models: (A) Independent Equiprobable; (B) Cooperative Equiprobable; (C) Independent 1 Slow Step; (D) Cooperative 1 Slow Step; (E) Cooperative 2 Slow Steps.

How can we glean insight about the process kinetics, and can we use a probabilistic framework to do so? Here we analyze the probability of observing reprogramming as a function of progress in the reprogramming protocol. Figure 6 shows the probability of a cell reprogramming in each model as a function of time after individual events occur. Reprogramming probability trajectories were computed using each of the three epigenetic events studied in the previous section, represented in green (DNA), red (H3K27) and blue (H3K4) curves; also included is the probability of reprogramming as a function of time after the beginning of induction, the black curve.

It is interesting to note that the black curves of Figure 6 have a basic resemblance to recent experimental results. Our data show cells starting to reprogram after just a few days and, depending on the model, the majority of cells has reprogrammed after 2 weeks (Independent Equiprobable model) or after 10–17 weeks (Cooperative 1 and 2 slow steps model, respectively). Data collected by Hanna and co-workers [20] show that 90% of wells seeded with single cells generate reprogrammed colonies after 16 to 18 weeks. The shape of the curve of percentage of cells reprogrammed as a function of time in our models is also similar to that measured experimentally.

The data for the Independent Equiprobable model (Figure 6A) shows the three lines for the three events being overlaid. This makes sense because no modification is more likely than the others thus; they signal the same level of advancement along the induction pathways. The fact that the lines are overlaid is also a basic indication of soundness of our simulations and their statistical convergence. The data for all three Cooperative mechanisms show a spread of the trajectories corresponding to the order in the mechanism (Figure 6B, D, and E). This makes intuitive sense, because the cooperative models have a strongly preferred ordering of events.

The results also reveal information about limiting kinetic aspects. The plot of the Cooperative 1 Slow Step model (Figure 6D) shows what the limiting step is. The large difference between the red and green line shows that the probability of observing reprogramming dramatically increases once H3K27 methylation is lost, the slow step. This is in contrast to the negligible change observed after we observe loss of DNA methylation (the green line is very similar to the black line).

The theme that slow steps can be revealed in reprogramming trajectories is also apparent in the Independent 1 Slow Step model (Figure 6C). While there is no order for this model, all successful reprogramming pathways must traverse a slow step that is essential – to lose H3K27 methylation. This corresponds to a modest acceleration in reprogramming trajectories once they have accomplished the slow step. Thus, a separation of scales may result from a slow step whether or not there is obligate ordering in the pathway. Figure 6C shows a slow step that tends to happen late because it is slow but not because the biochemistry *requires* that it occur after other epigenetic changes. Figure 6B, C, and E correspond to cases where the ordering is essentially obligate due to the modeled biochemistry. The common assumption that late events are required to occur after some early event is not always appropriate and may lead to incorrect conclusions, such as that it is not worth speeding up a late event when, in fact, it is.

Finally, from this data it is also possible to understand some of the biological principles that might give rise to the proposed views of elite or stochastic iPSC generation. For example, in Figure 6D, the state cells are in is an important determinant of reprogramming time. Cells that have already lost H3K27 methylation have an approximately 80% probability of reprogramming within 15 days; for cells that retain H3K27 methylated the probability drops to 15%. Cells that have lost H3K27 methylation could thus be construed as an elite subpopulation that is closer to reprogramming. Similarly, Figure 6E cells that lost DNA methylation (green line) have a 50% probability of reprogramming at around 30 days, whereas for the cells at the initial state 50% requires almost double that time. These results show that one way that elite-type results can be explained is by the existence of subpopulations of cells that overcome one (or several) of the low probability reactions on the way to reprogramming earlier than others.

Regarding these last observations, one key point is that all the cells in these simulations start with the same initial conditions (as described in Methods), yet, at any given time after induction, some will have had that reaction happen and some not. Therefore, stochastic processes acted upon a population that was homogeneous at time of induction and created a subgroup that can be said to have elite-like properties. The same results are obtained when cellular populations are already heterogeneous at the time of induction as shown in the next section.

#### Effects of pre-existing population variation

**Figure 7:**
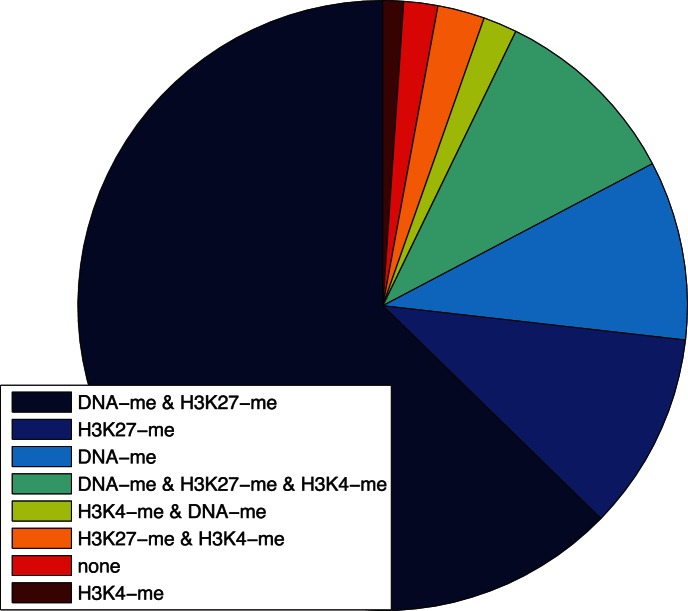
Population distribution. Distribution of states of the *NANOG* promoter in an equilibrium population of cells in the Independent Equiprobable model. A cell counts for a state if it has one of the gene copies in that state.

The work described up to this point in the paper reports on simulations that all began from the same uninduced starting point. Stochastic variation led to differences in simulated behavior across a population of initially identical cells. Even with such variation, key features of the kinetic pathway leading to full induction produced distinguishing features in the overall reprogramming dynamics of the populations. In this section we examine how pre-existing variation of uninduced cells can affect reprogramming dynamics. Simulations were made of 1500 cells with the Independent Equiprobable model and stochastic dynamics but without the inducing factors. The resulting population represents an equilibrium distribution expected prior to application of each induction protocol. The distribution shown in Figure 7 indicates that the population is not uniform. While most of the population is in the starting low state with each of the corresponding epigenetic marks set accordingly (H3K27 methylated, DNA methylated, and H3K4 demethylated), a significant number of cells have one of those marks changed in at least one of the two copies of the *NANOG* gene, and a much smaller number has two or even all three changed in at least one of the copies. The cells occupy this distribution of states due to finite, non-zero rates for flipping epigenetic marks and flipping them back. Stochastic events are responsible for which cells are in which state at any point in time. Once a steady-state distribution is reached, individual cells continue to change state, but the distribution is invariant. Thus, whereas stochastic events drive the system to its steady state, the steady-state distribution is deterministic and a characteristic of the modeled cell population.

**Figure 8:**
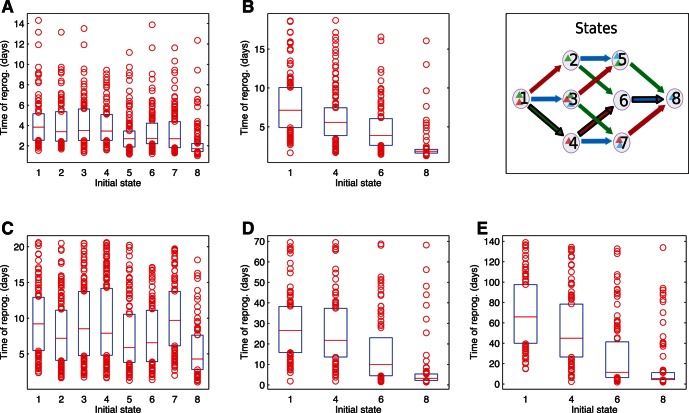
Reprogramming time as function of initial state. Time of reprogramming on the vertical axis. Red line is the median, blue box encompasses all points from 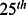 to 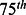 percentiles of the distribution. Initial state prior to induction in the horizontal axis numbered as indicated in the respective panel. Also, the black contour on the arrows represents the order in the cooperative mechanisms (in those models, only such states were used as initial conditions for these simulations). Models in each panel: (A) Independent Equiprobable; (B) Cooperative Equiprobable; (C) Independent 1 Slow Step; (D) Cooperative 1 Slow Step; (E) Cooperative 2 Slow Steps.

We hypothesized that the different subpopulations in the steady-state distribution could have different reprogramming dynamics, because some were further along the reprogramming pathway than others. Sharp differences in reprogramming time could give the appearance of an elite subpopulation especially primed for reprogramming. Fundamentally, however, the cells are equally capable of interconverting among the same set of states, and emerging differences are due to the state each cell happened to be in at the time the induction protocol was initiated. To explore the effect of pre-existing states on reprogramming dynamics, each of the eight substates was used to start sets of simulations under induction conditions. Simulations were run for all models, and results for the distribution of reprogramming times are given in Figure 8. Distinct subpopulations can have significantly different reprogramming times. This is especially true of the Cooperative model variants and particularly those with slow steps. Subpopulations starting further along the reprogramming pathway tended to complete the process more quickly than those beginning more distant from the final state. When one or more slow steps was present, substates after the last slow step reprogrammed much faster than those before. While terms such as "elite" may be applied to these subpopulations to indicate that they respond more quickly to induction protocols than other cells, for the case described here all cells are equally capable of reprogramming. The faster time scale available to these cells suggests it may be advantageous to isolate and induce only them, or even to search for methods to accelerate slow steps either to prepare cells for induction or to apply concomitant with induction.

#### Population dynamics during reprogramming

**Figure 9:**
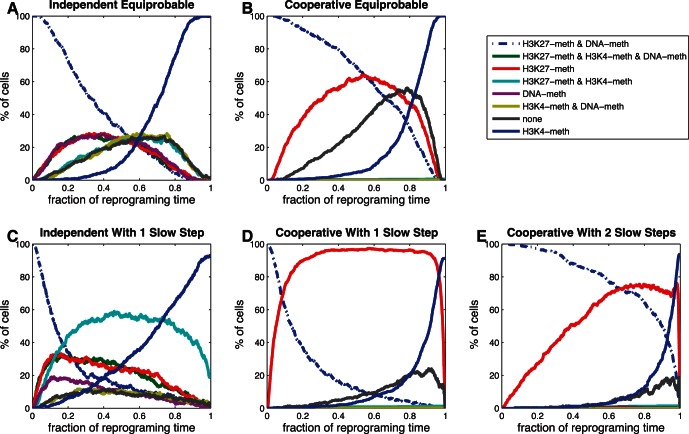
Analysis of fractional state occupancies. The horizontal axis represents fraction of reprogramming time, the vertical axis represents the percentage of cells that has one or two copies of the *NANOG* gene in the state corresponding to the curve legend. Models: (A) Independent Equiprobable; (B) Cooperative Equiprobable; (C) Independent 1 Slow Step; (D) Cooperative 1 Slow Step; (E) Cooperative 2 Slow Steps.

We reprocessed our induction simulation data to examine the timing of the progression through various states during the reprogramming protocols. Because cells simulated with the same model exhibited a wide range of reprogramming times, we adopted the practice of normalizing the time axis of each cell trajectory by its ultimate reprogramming time, which produces a population of trajectories in terms of the relative time 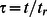 where 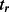 is the absolute reprogramming time for the current cell trajectory (thus, 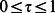). Averaging across all cells in a given model as a function of 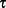 produced the relative population trajectories in Figure 9.

Figure 9A shows that the Independent Equiprobable model's population starts in an epigenetic state corresponding to the adult cell state. As cells leave that state they populate states 2, 3, and 4 (nomenclature of Figure 8) with equal probability; at later relative reprogramming time cells populate states 5, 6, and 7 (with two of the three changes) before moving on to the final epigenetic state and reprogramming at 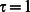. While the population occupancies display a steady, progressive accumulation of epigenetic changes toward reprogramming on average, individual cells can and do make temporary steps backwards before ultimately reprogramming. In Figure 9A, the grouping of the two sets of three lines that refer to states of equal distance from the starting state is, in fact, what we would expect to observe in conditions of statistically converged simulations.

The introduction of a slow step (Figure 9C) leads to somewhat different population dynamics with high population accumulation before the slow steps and population after the slow step generally advancing quickly to the final state. Cooperative mechanisms essentially only populate the preferred pathway (Figure 9B, C, and E). When slow steps are introduced, the resulting bottleneck creates large buildups of the corresponding intermediate (Figure 9C and E).

### Opportunities for Accelerating Stem Cell Induction Dynamics

**Figure 10:**
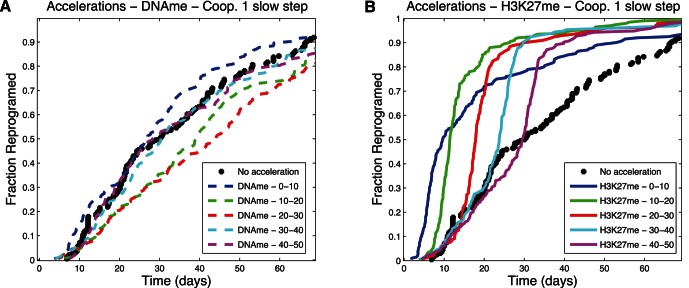
Acceleration plots – Cooperative 1 Slow Step. Fraction of cells that has reprogrammed as a function of time. (A) Accelerating the reaction of loss of DNA methylation. (B) Accelerating the reaction that leads to loss of H3K27 methylation. Different times of acceleration, corresponding to percentages of total time, correspond to the lines identified the in the legend.

**Figure 11:**
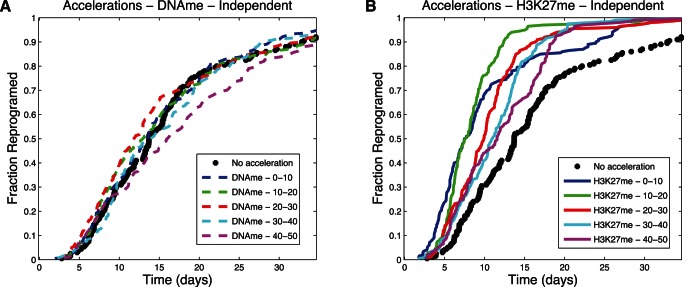
Acceleration plots – Independent 1 Slow Step. Fraction of cells that has reprogrammed as a function of time. (A) Accelerating the reaction of loss of DNA methylation. (B) Accelerating the reaction that leads to loss of H3K27 methylation. Different times of acceleration, corresponding to percentages of total time, correspond to the lines identified the in the legend.

**Figure 12:**
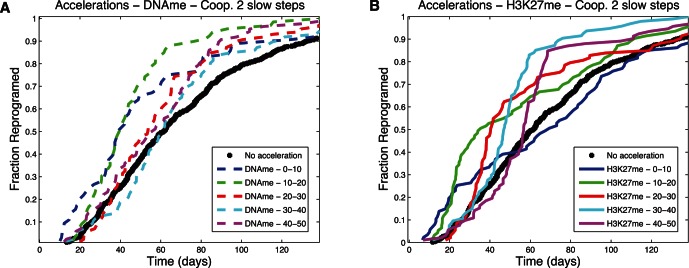
Acceleration plots – Cooperative 2 Slow Steps. Fraction of cells that has reprogrammed as a function of time. (A) Accelerating the reaction of loss of DNA methylation. (B) Accelerating the reaction that leads to loss of H3K27 methylation. Different times of acceleration, corresponding to percentages of total time, correspond to the lines identified the in the legend.

The analysis of reprogramming dynamics from the simulation showed the presence of bottlenecks occurring before slow steps. Here we explore the effect of accelerating individual reaction steps on the overall reprogramming rate. For the purposes of this study, we artificially increased the rate of the selected reaction by increasing the associated reaction rate by 10 fold for a brief period of time corresponding to 10% of the total protocol time for the given model. Figures 10, 11, and 12 present results for accelerating each of two different reaction steps (loss of DNA methylation and loss of H3K27 methylation) in three different models (Cooperative 1 Slow Step, Independent 1 Slow Step, and Cooperative 2 Slow Steps) with varied time of pulse application. The results show dramatic increases in overall reprogramming efficiency when the slow steps were accelerated but not when a fast step was accelerated (Figure 10B *vs.* A and Figure 11 B *vs.* A). Interestingly, the overall efficiency improvement was relatively insensitive to the time of pulse application. This can happen because all cells that haven't reprogrammed are trapped in a particular state (this can be seen, for example, in Figure 9D). Pulses introduced concomitant with the start of the induction protocol were somewhat less efficient because too few cells had left the starting state and were yet trapped at the slow step by the time the pulse had ended (Figure 10B). Likewise pulses applied when many cells had already passed the slow step (Figure 12A) or were waiting at a different non-accelerated step (Figure 12B) were also less effective.

Understanding the influence of mechanisms and kinetics in accelerating particular reactions is especially relevant, considering the evidence that suggests some of the cells that do not reprogram in the initial weeks of the protocols are relatively stable in partially reprogrammed states [29], [50]. Characterization of these cells revealed that the promoters of key genes of the reprogramming circuitry remained heavily methylated and some of the necessary histone modifications have not happened. Regarding our work, Figure 9D shows that a dominant slow step can cause cells to remain in the same state most of time until reprogramming, and the black line in Figure 6D shows that only 20% of cells collected at day 14 would have reprogrammed; by implication (Figure 9D), those that did not reprogram would not have done so because of the slow step keeping them in their unreprogrammed state. As illustrated in Figure 10B, had these cells been made more likely to traverse the slow step in time, the overall efficiency of the process could have been greatly increased. Taken together, these observations suggest one of the reasons for low efficiency may be related to particularly slow kinetic steps in an ordered mechanism. It also suggests that identifying particular steps that cells struggle to overcome (because they are inherently slow or rare) and accelerating them, as shown in Figure 10B, may limit the loss of efficiency due to partially reprogrammed cells.

### Conclusion

This work used computational modeling to examine induced cellular reprogramming of differentiated adult cells to stem cells. Our study focused on the relationship between the individual biochemical steps underlying reprogramming and the dynamics of the overall process. The use of stochastic models revealed the distributed behavior of the populations of cells. Much of the mechanistic biology of the inverse differentiation process leading to induced pluripotent stem cells is yet to be discovered. Therefore, our model, despite an improvement on previous modeling work, can at best be only an approximation of the actual process of interest. In the absence of experimental measurements that report kinetic values or definite topologies, this and other models of the cellular pluripotency network are likely to be missing features that will be shown to play a role and rate parameters and mathematical dependencies that can be improved as further studies report their results. Moreover, by their very nature, models can never be proven correct; at best they can be consistent with a collection of available data. Models are nevertheless tremendously useful, including as a basis for understanding the relationship between network construction and overall functional operation. The model and parameterization we use are consistent with the basic experimental observations (bistability, inducibility, stochasticity, and the basic shape and timing of the reprogramming curves) and can be used to understand some of the relationships of the reprogramming protocols we focus on in this work. Here we examined how detailed kinetic properties, such as the existence and placement of slow steps and the degree of cooperative ordering of kinetic events, affects the distribution of overall reprogramming dynamics for a population of cells. Models are also fundamental substrates for engineering and design. Here we evaluated approaches to accelerating cellular reprogramming using a modeling framework.

A relatively simple set of models is consistent with the observed properties of bistability, inducibility, and variability. Our models share cooperativity in three places (epigenetic cooperativity linked to transcription factor binding, dimerization of NANOG, and cooperative promoter binding of SOX2 and OCT4), the first of which is most important for bistability.

The broad distribution of reprogramming times that occurs in the stochastic modeling simulations, particularly when there are slow steps, can make it difficult to ascertain whether all cells are eventually capable of reprogramming, with some just taking longer than others. A multi-step reprogramming pathway can lead to subpopulations that are further along the path than others and that tend to reprogram more quickly. Here, all cells were genetically identical and capable of interconverting among all states, yet with fixed times allotted for reprogramming, only a subset complete the process. Given sufficient time all do. This behavior appears consistent with recent experiments [20].

Results show that slow steps, because they have a wide range of waiting times, introduce large variance in overall reprogramming times. Results also demonstrate that observing a preferred ordered of kinetic events does not itself allow a conclusion to be drawn about its source. While it is tempting to imagine an obligate ordering of events, for example through cooperativity and other mechanisms, the presence of slow and fast steps can also explain the observations.

We show that, even when simulated to account for biochemical stochastic dynamics, cooperative mechanisms lead to unpredictability of outcome being reduced as the cells move along the path of necessary modifications to reprogram. This is in contrast with the prediction for independent mechanisms, where observations of modifications are less informative about reprogramming times.

It's known that different types of cells have different reprogramming potential [51]. In our work we examine the influence of the existence of subpopulations of cells in reprogramming dynamics. We studied the scenario where cells are all equal at induction but accrue differences due to stochastic events, and the scenario where there are subpopulations of similar cells with different epigenetic features. In both cases we illustrate how cellular state, acquired or pre-existing, can lead to elite-type behavior and cause significantly different reprogramming times. This, in our predictions, is especially true for the cooperative mechanisms and particularly when dominated by slow steps. Slow steps can create kinetic barriers to reprogramming that may create the appearance of elite behavior.

Kinetics and mechanisms also have an important impact when the desire is to accelerate reprogramming protocols. If the process is dominated by a single slow reaction, then accelerating that reaction is the key to improving efficiency. If the process is dominated by several slow steps, then accelerating only one of them has a smaller effect and one might consider having to accelerate several of them – in which case the order of intervention for acceleration becomes important. Recent studies suggest that SOX2 has a specific time window of action [52]. In light of our results, this is consistent with the hypothesis of a mechanistically ordered system of modifications on the path to reprogramming. Further, it raises the possibility that the action of this transcription factor is one of the limiting steps for reprogramming. Naturally, only with further experimentation can this be confirmed. Possible experiments include inhibiting specific steps to determine whether those steps are part of an ordered mechanistic chain of events or an unordered process. If a step is part of an ordered necessary path, then inhibiting it will trap cells in that state; on the other hand, if a step is part of an unordered mechanism then inhibiting it will not impede progress along other necessary modifications. Similar to the work done with SOX2 there are also recent indications that the timing of action for promoting DNA demethylation is an important feature [53], and recent work showing that cells can become trapped due to-non completion of this step [29]. Once again, our work suggests that one explanation for these observations might be that this epigenetic modification is part of an ordered cooperative mechanism of changes with more than one slow step. Given these observations and recent developments that indicate the possibility of substituting factors for reprogramming with small molecules [54] (which can easily be supplied at different times), as well as recent work describing screening approaches that were used to inhibit the action of specific kinases important for the reprogramming process [55], we suggest that experiments with time-dependent screening of inhibition or acceleration of particular steps might be used to understand not just what steps are limiting, but also the mechanistic dependencies between the several key steps of the reprogramming process.

Besides suggesting a different take on the elite *versus* stochastic framework proposed by Yamanaka [19], we believe that work presented here will help increase the efficiency of cellular programming. Indeed, as illustrated, certain types of data about the times of milestone events and subsequent analysis can reveal kinetic and mechanistic information about the process of cellular reprogramming. We also suggest that time-dependent experiments with acceleration and inhibition of certain steps can be used to identify limiting and mechanistic relations. When this information is obtained, it is possible that accelerating those most sensitive points in the process at the appropriate times, or focusing on cells that have passed these barriers to the final stage, might prove to be effective in improving the efficiency of iPSC generation protocols. Mechanistic information about the process is also needed to improve safety of potential future applications. Indeed, alongside the promise that induced pluripotent stem cells hold for fundamental research and for medical applications, there is also recent evidence of unstable or imperfect reprogramming [56], [57]. This work underscores the point that if the efficiency and safety of these protocols is to be improved, then a more mechanistic understanding may be useful. The work done here also points to the necessity of single-cell measurements, because measurements of population averages do not reveal the detailed mechanistic and kinetic intricacies of the process.

## Supporting Information

File S1 **The zip file contains all five models used in this work, in their matlab2008 “sbproj” format.** Models are: (Model S1 in File S1) Independent Equiprobable, (Model S2 in File S1) Cooperative Equiprobable, (Model S3 in File S1) Independent 1 Slow Step, (Model S4 in File S1) Cooperative 1 Slow Step, (Model S5 in File S1) Cooperative 2 Slow Steps.
(ZIP)
